# The 5-Phosphatase SHIP2 Promotes Neutrophil Chemotaxis and Recruitment

**DOI:** 10.3389/fimmu.2021.671756

**Published:** 2021-04-19

**Authors:** Melina Michael, Barry McCormick, Karen E. Anderson, Utsa Karmakar, Matthieu Vermeren, Stéphane Schurmans, Augustin Amour, Sonja Vermeren

**Affiliations:** ^1^ Centre for Inflammation Research, Institute for Regeneration and Repair, The University of Edinburgh, Edinburgh, United Kingdom; ^2^ Signalling Programme, The Babraham Institute, Cambridge, United Kingdom; ^3^ Centre of Regenerative Medicine, Institute for Regeneration and Repair, The University of Edinburgh, Edinburgh, United Kingdom; ^4^ Laboratory of Functional Genetics, GIGA Research Centre, University of Liège, Liège, Belgium; ^5^ Adaptive Immunity Research Unit, GlaxoSmithKline, Stevenage, United Kingdom

**Keywords:** neutrophil, chemotaxis, recruitment, PI3K, SHIP2, SHIP1, lipid second messenger

## Abstract

Neutrophils, the most abundant circulating leukocytes in humans have key roles in host defense and in the inflammatory response. Agonist-activated phosphoinositide 3-kinases (PI3Ks) are important regulators of many facets of neutrophil biology. PIP3 is subject to dephosphorylation by several 5’ phosphatases, including SHIP family phosphatases, which convert the PI3K product and lipid second messenger phosphatidylinositol 3,4,5-trisphosphate (PIP3) into PI(3,4)P2, a lipid second messenger in its own right. In addition to the leukocyte restricted SHIP1, neutrophils express the ubiquitous SHIP2. This study analyzed mice and isolated neutrophils carrying a catalytically inactive SHIP2, identifying an important regulatory function in neutrophil chemotaxis and directionality *in vitro* and in neutrophil recruitment to sites of sterile inflammation *in vivo*, in the absence of major defects of any other neutrophil functions analyzed, including, phagocytosis and the formation of reactive oxygen species. Mechanistically, this is explained by a subtle effect on global 3-phosphorylated phosphoinositide species. This work identifies a non-redundant role for the hitherto overlooked SHIP2 in the regulation of neutrophils, and specifically, neutrophil chemotaxis/trafficking. It completes an emerging wider understanding of the complexity of PI3K signaling in the neutrophil, and the roles played by individual kinases and phosphatases within.

## Introduction

Neutrophils are the most abundant circulating leukocytes in humans. These polymorphonuclear phagocytes provide a first line immune response against infection by invading pathogens and play a key role in the development of the inflammatory response. Neutrophils express a range of G protein coupled chemoattractant/chemokine receptors with the help of which they detect, and quickly react to gradients of chemoattractants, e.g. bacterial peptides. This underpins their ability to leave the blood stream and move directionally (chemotax) towards sources of chemoattractant. Once neutrophils reach the sites of inflammation, they deploy a range of effector functions including phagocytosis, degranulation, production of reactive oxygen species (ROS), and the release of neutrophil extracellular traps (NETs) to eliminate pathogens (1).

Amongst the proximal enzymes activated downstream of the chemoattractant receptor-ligand interaction is phosphoinositide 3-kinase (PI3K), which generates the lipid second messenger phosphatidylinositol(3,4,5)trisphosphate (PIP3) by phosphorylating the D3 position of the inositol ring of phosphatidylinositol(4,5)bisphosphate (PI(4,5)P2), an integral component of the inner leaflet of the plasma membrane (2). Neutrophils express all four isoforms of agonist-activated PI3K. PIP3 causes the recruitment to the plasma membrane and activation of PI3K effectors, many of which are expressed in the neutrophil (3). The localization of PIP3 at the leading edge is one of the earliest molecular events in neutrophil chemotaxis (4, 5), thought to be important for their ability to polarize and subsequently migrate directionally towards a source of chemoattractant.

PI3K activity is counteracted by phosphatases which hydrolyze the short-lived PIP3. As a major 3-phosphatase, phosphatase and tensin homolog (PTEN) converts PIP3 back to PI(4,5)P2, while the hematopoietic cell-restricted SHIP1 is thought to be a major 5-phosphatase in leukocytes that dephosphorylates PIP3 to form PI(3,4)P2, a lipid second messenger in its own right that shares some effectors with PIP3 (6). Global PTEN-deficiency is embryonic lethal (7), but SHIP1-deficient mice are viable and fertile, however, they exhibit a shortened lifespan that is thought to be due to leukocyte infiltration of the lungs (8, 9). Both PTEN and SHIP1-deficient neutrophils were previously described; PTEN knockout neutrophils are characterized by increased PIP3 (10), enhanced ROS production when stimulated with fMLF, increased ruffling and sensitivity to chemoattractants, a minor directionality defect (11), and a lengthened lifespan (12), while SHIP1 knock-out neutrophils display reduced ROS production  (13) and augmented apoptosis (14). SHIP1-deficient neutrophil spread extensively on the substratrum, and in response to chemoattractant stimulation fail to polarize and chemotax efficiently towards a chemoattractant (15).

In addition to SHIP1, neutrophils also express its ubiquitous isozyme, SHIP2, the function of which in the neutrophil remains uncharacterized. In this study, we describe the analysis of neutrophils from a mouse (here called Ship2^Δ/Δ^) that carries a small deletion in the SHIP2 catalytic domain which renders it catalytically dead (16). We demonstrate that SHIP2 is an important regulator of neutrophil chemotaxis *in vitro* and of neutrophil recruitment to sites of sterile inflammation *in vivo*, whereas other neutrophil functions remain essentially intact. While we do not detect differential PI3K activity when using PKB phosphorylation as an indirect read-out, PI(3,4)P2 was found to be reduced.

## Materials and Methods

Unless otherwise specified, materials were acquired from Sigma Aldrich (Gillingham, UK). All reagents were of the lowest available endotoxin level. Tissue culture media and buffers were from Gibco (Thermo Fisher Scientific, Loughborough, UK). Percoll was from GE Healthcare (Amersham, UK).

### Antibodies

Anti-HSP90 (clone 3H3C27), anti-SHIP1 (clone PICI-A5), FITC-conjugated rat anti-mouse GR1 (clone RB6-8C5), PE-conjugated rat anti-mouse/human CD11b (clone M1/70), PE/Cy7-conjugated rat anti mouse/human CD45 (clone 30-F11), PerCP/Cy5.5-conjugated rat anti F4/80 (clone BM8), APC-conjugated rat anti-B220 (clone RA3-6B2), PE/Cy7-conjugated rat anti CD3 (clone 17A2) and pacific blue-conjugated rat anti-LY6G (clone 1A8) were from BioLegend (London, UK); anti-PTEN (clone D4.3), anti-PKB (clone 11E7), anti-PKB T308 (clone C25E6) and anti-PKB S473 (clone D9E) were from Cell Signaling Technology (London, UK). Rabbit IgG (I8140) was obtained from Sigma. Anti-SHIP2 (AF5389) and PE-conjugated rat anti-CD64 (clone FAB20741P) were from R&D Systems (Abingdon, UK) and biotinylated anti-PI(3,4)P2 (z-B034) was from Echelon Biosciences (Salt Lake City, UT, USA); streptavidin-AF647, AF488-conjugated phalloidin, AF568-conjugated phalloidin, and secondary antibodies anti-rat AF488, anti-rabbit AF568 and anti-rabbit AF488 were obtained from Thermo Fisher Scientific (Loughborough, UK).

### SHIP2 Mouse Model

Ship2^Δ/Δ^ mice (16) were housed in individually ventilated cages in a specific opportunistic pathogen-free small animal barrier unit at the University of Edinburgh. After backcrossing for eight generations to C57Bl/6 background, Ship2^Δ/Δ^ and wild-type controls were derived by Ship2^Δ/+^ intercrosses. Sex and age-matched mice were used in experiments. All animal work was approved by the University of Edinburgh Animals Welfare and Ethical Review Body and conducted under the control of the U.K. Home Office (PPL 60/4502 and PFFB 42579).

### Neutrophil Preparations

Bone marrow-derived neutrophils were prepared from the tibias and femurs of age and sex-matched mice on a discontinuous percoll gradient as previously described (17), using endotoxin-free reagents throughout. Neutrophil preparations typically reached ~70% purity as assessed by Diff-Quik-stained cytocentrifuge preparations. Unless otherwise stated, experiments were performed in Dulbecco’s PBS supplemented with Ca^2+^ and Mg^2+^, 1g/L glucose and 4mM sodium bicarbonate.

### Degranulation

Lactoferrin release was assayed by making use of an antibody directed to human lactoferrin that had previously been shown to cross-react with mouse protein as described (18, 19).

### Phagocytosis

0.8 μm diameter latex beads were opsonized with polyclonal rabbit IgG as per manufacturer’s instruction. TNF (1000 U/mL) and GM-CSF (100 ng/mL)-primed neutrophils (R&D Research, Abingdon UK) were stimulated with IgG-opsonized latex beads at a ratio of 5:1 for 20 mins at 37°C. Cells were allowed to adhere onto coverslips for 1h on ice and then fixed with 2% ice-cold paraformaldehyde (PFA) for 10 minutes. Adherent latex beads were labelled with anti-rabbit AF568, prior to cell permeabilization with 0.1% Triton X-100, labelling of all latex beads with anti-rabbit AF488; cells were mounted with ProLong Gold (Thermo Fisher Scientific, Loughborough UK). Cells were viewed using an Evos cell imaging system (Thermo Fisher). The percentage of cells that had internalized beads, and internalized beads/cell were recorded.

### Analysis of ROS Production

ROS production was detected indirectly by measuring chemoluminescence production by 5x10^5^ neutrophils/well using luminescence-grade 96-well plates (Nunc, Thermo Fisher Scientific Loughborough, UK) in a Cytation plate reader (BioTek, Swindon, UK) as described (17, 20) with neutrophils incubated with 150μM luminol and 18.75U/ml horseradish peroxidase. Data output was in light units/second.

### Chemotaxis

Chemotaxis was analyzed with neutrophils resuspended in HBSS supplemented with 15mM HEPES (pH 7.4) and 0.05% fatty acid and endotoxin-free BSA. For integrin-dependent chemotaxis, neutrophil migration on a glass bridge was monitored by time lapse-imaging for 30 minutes in Dunn chambers (Hawksley, Lancing, UK). Dunn chambers were assembled as previously described (21) with 300nM fMLF as the chemoattractant. For integrin-independent chemotaxis, neutrophils were mixed with a 3D collagen matrix (A1048301, Roche Diagnostics, Mannheim, Germany), which was prepared as per manufacturer’s instructions, and left to polymerize in a humidified incubator at 37°C at 5% CO_2_ before cells were allowed to migrate towards 300nM fMLF in Chemotaxis μ-slides (Ibidi, Martinsried, Germany). Images were acquired on a Leica IRB inverted microscope with temperature-controlled chamber, automated stage (Prior, Cambridge UK), Orca camera (Hamamatsu, Welwyn Garden City, UK) and Micromanager image acquisition software (Fiji). Paths of individual cells were tracked using the manual tracking plug-in into Image J and tracks analyzed using the Chemotaxis Tool (Ibidi) plug-in into Image J as described (19).

### Adhesion Under Laminar Flow

Ibidi VI^0.4^ flow chambers that had been coated with recombinant murine (rm) ICAM-1 (15μg/mL), rmE-selectin (20μg/mL; both Biolegend) and rmCXCL1 (15μg/mL; Biotechne, Minneapolis, MN, USA) were perfused with bone marrow derived neutrophils at 37°C to deliver a constant sheer stress of 1 dyne/cm^2^ using a syringe pump (Legato 200; KD Scientific, Holliston, MA, USA) (20). Cell adhesion under flow was recorded by time-lapse imaging (2.5 images/s) for 1 minute at 1, 5, 10 and 15 minutes after starting flow with a x20 phase contract objective using a Leica IRB inverted microscope (Leica, Milton Keynes, UK). Firmly adherent cells were counted using ImageJ.

### Reconstitutions

Cohorts of female C57Bl/6 mice were subjected to two doses of irradiation (4.5Gy) 3 hours apart, and reconstituted the next day by tail vein injection of 4 x 10^6^ T-cell depleted (CD3ε microbead kit (Militenyi Biotech, Surrey, UK) bone marrow cells from Ship2^Δ/Δ^ mice or wild-type littermates. Following irradiation mice were offered enrofloxacin (Bayer, Cambridge, UK) in their drinking water for 4 weeks. Reconstitution of the hematopoietic system in bone marrow chimeras was confirmed by analyzing test bleeds by flow cytometry, comparing ratios of B cells, myeloid cells and neutrophils in chimera to those in wild-type control bloods. Control and Ship2^Δ/Δ^ bone marrow cells were equally able to reconstitute irradiated recipients (not shown).

### Blood Cell Counts

10-12-week-old control and Ship2^Δ/Δ^ littermates were subjected to cardiac puncture under terminal isofluorane anaesthesia with confirmation of death by cervical dislocation. Blood was collected into EDTA-coated vacutainers (Sarstedt, Nümbrecht, Germany). Erythrocyte counts were obtained from an automated Alpha VET cell counter (Nihon Kohden, Surrey, UK); leukocyte markers were labelled and leukocyte numbers obtained by volumetric counting using an Attune NxT flow cytometer (Thermo Fisher).

### Models of Acute Sterile Inflammation

To induce thioglycollate peritonitis, mice were intraperitoneally administered 20 ml/kg matured Brewer’s thioglycollate (BD Biosciences; Wokingham, UK). LPS-induced acute lung inflammation (ALI) was performed as previously described (20) by administering 1µg *E.coli-*derived LPS (serotype O127:B8, Sigma) in 50µl sterile saline intratracheally. 15 minutes prior to being sacrificed, mice received 3µg PE/Cy7 labelled anti-CD45 in 100µl sterile saline to label all intravascular leukocytes. Lavage cells were labelled with FITC-anti-GR1 and APC-anti-CD11b and analyzed by flow cytometry to calculate total neutrophils numbers (GR1^high^, CD11b^+^).

### Immunoblotting for PI3K Activity

Neutrophils in PBS^++^ were pre-warmed for 5 min at 37°C prior to being stimulated as indicated with fMLF or vehicle for indicated times. Cells were lysed in ice-cold 20mM Tris-HCl pH 7.5, 150mM NaCl, 1mM EDTA, 1mM EGTA, 1% Triton X-100, 2.5mM Na pyrophosphate, 1mM β-glycero-phosphate, 1mM Na orthovanadate, 0.1mM PMSF and 10µg/ml of each antipain, aprotinin, pepstatin A and leupeptin for 5 minutes. Clarified lysates were subjected to SDS-PAGE, and proteins transferred to Immobilon membrane (Merck Millipore, Darmstadt, Germany) and subjected to Western blotting with phosphospecific antibodies directed against PKB as well as a loading control (HSP 90).

### PIP3 Measurements

Neutrophils were prepared for PIP3 detection essentially as described (22). Neutrophil aliquots (1 x 10^6^ cells in 135 μl) were stimulated with pre-warmed 865 μl fMLF (final concentration 10μM) or vehicle. At specified times 5 ml of ice-cold initial organic mix (CHCL3:MeOH, 1:2 v:v) were added and sampled stored at -80°C until lipid extractions were performed in the presence of internal standards (d6-C18:0/C20:4-PIP3 (10ng) and –PI(4,5)P2 (100ng)) to correct for any variation in recovery. The analysis of inositol lipids was performed as previously described (22) using a QTRAP 4000 (AB Sciex) mass spectrometer. Data are shown as response ratios, calculated by normalizing the MRM targeted lipid integrated response area to that of a known amount of relevant internal standard. PIP3 response ratios were normalized to PIP2 response ratio to account for any cell input variability.

### Immunocytochemistry and Image Acquisition

Neutrophils were allowed to attach onto glass coverslips for 10 minutes prior to being stimulated with fMLP (1 μM final concentration) or vehicle. At indicated times, cells were fixed in 2% paraformaldehyde. PI(3,4)P_2_ immunostaining was performed essentially as described (23) with 0.5% saponin-permeabilized neutrophils being labelled with a biotin-conjugated primary antibody and streptavidin coupled AF647 as detection reagent. Samples were mounted and 8 images were acquired semi-automatedly using Zen software and a widefield Zeiss Observer with a 20x objective (Zeiss, Oberkochen, Germany); Confocal microscopy was performed using a Leica TCS SP8 microscope with a 60x objective and Lasx image acquisition software. For comparing signal intensities, all settings were kept constant between conditions and experiments.

### Image Analysis

Automated image analysis pipelines in CellProfiler (24) were used to determine cell size, brightness and polarization. Briefly, cells were segmented using nuclei (DAPI) and neutrophil-specific GR1 signals. In cells that had been thus identified signal intensity, intensity distribution and cell shape were then measured. Confocal images were processed with FiJi.

### Statistical Analysis

Statistical analysis was performed with Graph Pad Prism 8. Where data met the assumptions for parametric tests, two-tailed t-tests, paired t-tests or 2-way ANOVAs with multi-comparison post-hoc tests were performed; otherwise, the non-parametric Mann-Whitney test was applied. For kinetic experiments, the area under the curve was used for analysis. *p* values < 0.05 were deemed statistically significant.

## Results

**Figure 1:**
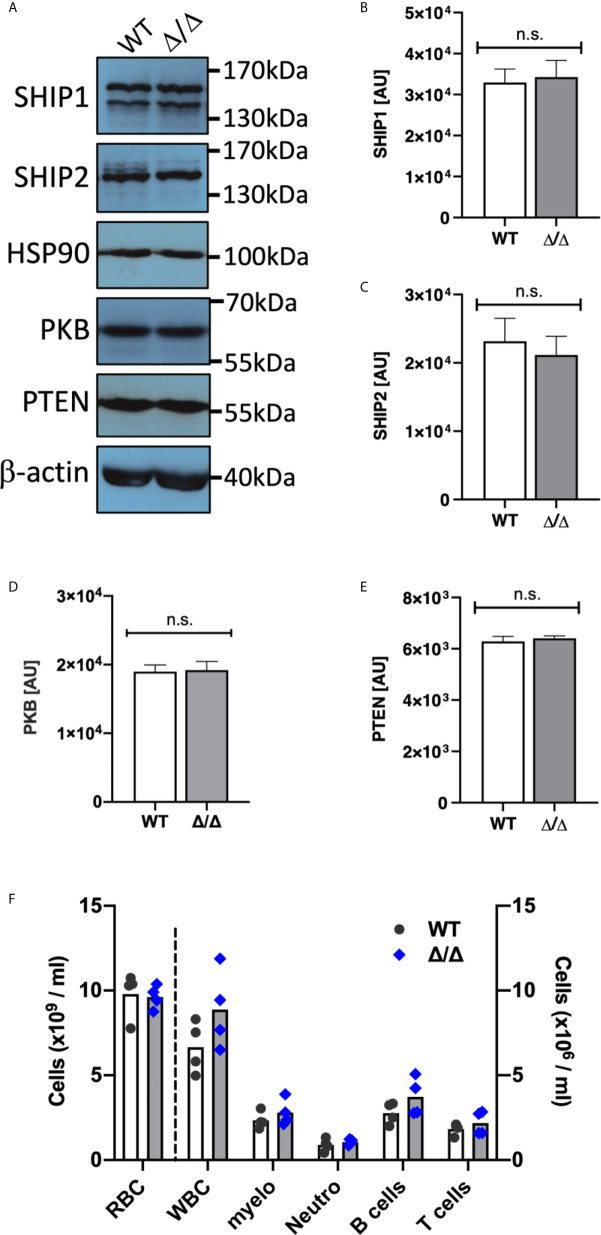
SHIP1/2 and PTEN expression is not affected in Ship2^Δ/Δ^ mice. Neutrophils from wild-type (WT) and Ship2^Δ/Δ^ (Δ/Δ) mice were tested for SHIP1, SHIP2, PTEN, PKB and loading control (HSP90, β-actin) expression. **(A)** Representative examples and **(B–E)** densitometry of 4 (PTEN, PKB, HSP90) or 5 (SHIP1/2, β-actin) separate experiments performed. Mean ± SEM are presented; AU, arbitrary units. n.s., not significant. **(F)** A comparison of blood cell counts between wild-type and Ship2^Δ/Δ^ mice. RBC, red blood cells; WBC, white blood cells; myelo, myeloid cells; neutro, neutrophils. Every symbol represents data obtained from one mouse. *p* values were determined by unpaired two-tailed t tests; differences did not reach significance.

Ship2^Δ/Δ^ mice carry a 57 amino acid deletion in their catalytic domain which renders SHIP2 catalytically dead (16). These mice share their characteristically short faces, small stature and leanness with a previously described SHIP2-deficient mouse (16, 25). We prepared bone marrow derived neutrophils from Ship2^Δ/Δ^ mice and matched wild-type controls and compared expression of the PIP3 phosphatases SHIP1, SHIP2 as well as the lipid phosphatase PTEN and the protein kinase PKB (also known as Akt). No differential expression was observed (**Figures 1A–E**). This contrasts with a prior observation in adipose and muscle tissue, where SHIP2^Δ/Δ^ protein expression was found to be significantly reduced (16).

### Lungs of Ship2^Δ/Δ^ Mice Are Not Infiltrated by Leukocytes

Ship1^-/-^ mice were characterized by a substantial increase in circulating myeloid cells in the peripheral blood (8, 9). They developed myeloid cell hyperplasia in the bone marrow and spleen from an early age and developed sterile inflammatory macrophage/neutrophil lung infiltration, which consequently caused >50% to die by only 10 weeks of age (8, 9). In contrast, blood cell counts were not affected in Ship2^Δ/Δ^ mice (**Figure 1F**). SHIP2-deficient and Ship2^Δ/Δ^ mice survived over 18 months (16, 25). Unchallenged Ship2^Δ/Δ^ mice housed in individually ventilated cages in our specific opportunistic pathogen free small animal unit did not display any signs of disease or distress. We used flow cytometry to analyze lung digests from 7-9-month-old mice, noting no obvious immune cell infiltrations in lungs of Ship2^Δ/Δ^ mice, nor splenomegaly (**Figure S1**), further supporting the notion that unchallenged Ship2^Δ/Δ^ mice are not prone to developing myeloid cell infiltration into their lungs, even at an advancing age.

### SHIP2 Regulates *In Vivo* Neutrophil Recruitment to Sites of Inflammation

**Figure 2:**
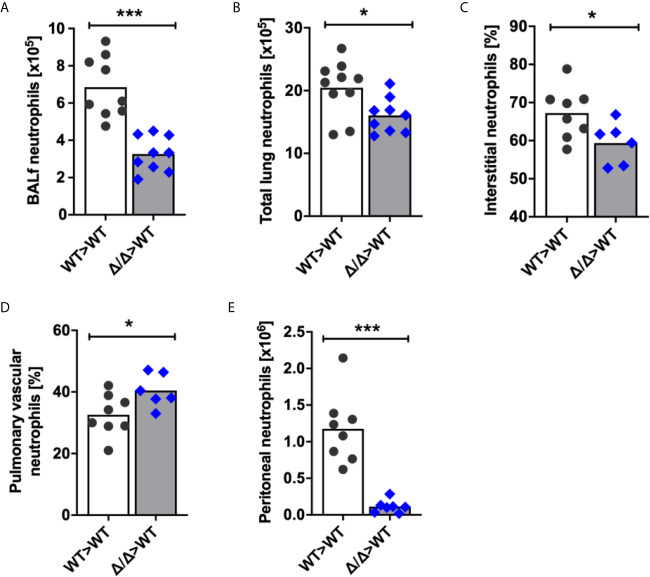
SHIP2 activity is required for neutrophil recruitment to sites of sterile inflammation. **(A–D)** Neutrophil recruitment in acute lung injury (ALI). ALI was induced by administering 1μg LPS in 50μl sterile saline intratracheally into 9 wild-type (WT) and Ship2^Δ/Δ^ (Δ/Δ) bone marrow chimeras (generated with 4 bone marrow donors per genotype). Neutrophil numbers retrieved from **(A)** bronchoalveolar lavages and from **(B)** lung digests are plotted. **(C, D)** Chimeras were i.v. administered fluorescently coupled anti-CD45 antibody prior to lavaging of saline-perfused lungs. Single-cell lung digests were then analyzed by flow cytometry. Percentages of **(C)** interstitial CD45 label-negative neutrophils and **(D)** pulmonary intravascular or partially transmigrated CD45 label-positive neutrophils are plotted. **(E)** Neutrophil recruitment in thioglycollate peritonitis. Peritonitis was induced by injecting 20ml/kg thioglycollate-containing broth into 8 wild-type and 7 Ship2^Δ/Δ^ bone marrow chimeras; the peritonea were flushed 2.5 hours later. Peritoneal neutrophil numbers are plotted. Experiments were performed on two separate days and results pooled in the graphs shown. Every symbol represents result obtained from one mouse, with means obtained indicated by bars; *p* values were determined by unpaired two-tailed t tests. *p < 0.05; ***p < 0.001.

To determine whether SHIP2 regulates neutrophil recruitment to the lungs upon inflicting an inflammatory challenge, we generated bone marrow chimeras and analyzed neutrophil recruitment in response to LPS-induced acute lung injury (ALI). We recovered a significantly decreased number of Ship2^Δ/Δ^ neutrophils compared to wild-type controls from bronchoalveolar lavages (BAL) of these chimeras (**Figure 2A**). In addition to examining BAL fluid, we also determined total lung neutrophil numbers in single cell digests of PBS-perfused lungs by flow cytometry. This identified reduced neutrophil counts in lungs from Ship2^Δ/Δ^>wt as opposed to wt>wt bone marrow chimeras (**Figure 2B**). Within the total lung neutrophils, we distinguished between circulating neutrophils that had firmly adhered to the vessel wall or partially transmigrated and those that were truly interstitial by labelling fully or partially intravascular neutrophils with a fluorescently conjugated anti-CD45 antibody delivered intravenously immediately prior to lung perfusion and tissue harvest. We observed fewer interstitial (anti-CD45^-^) and increased vascular (anti-CD45^+^) Ship2^Δ/Δ^ than wild-type control neutrophils (**Figures 2C, D**).

Neutrophil recruitment can be differentially regulated in a site- and stimulus-specific manner. For this reason, we also analyzed neutrophil recruitment in thioglycollate-induced peritonitis in Ship2^Δ/Δ^>wt and wt>wt bone marrow chimeras, again observing a substantial recruitment defect of Ship2^Δ/Δ^ neutrophils (**Figure 2E**).

Together, these experiments identified that Ship2^Δ/Δ^ neutrophil recruitment to sites of sterile inflammation is impaired, and suggested a reduced ability of Ship2^Δ/Δ^ neutrophils to extravasate.

### SHIP2 Regulates Neutrophil Chemotaxis and Directionality

**Figure 3:**
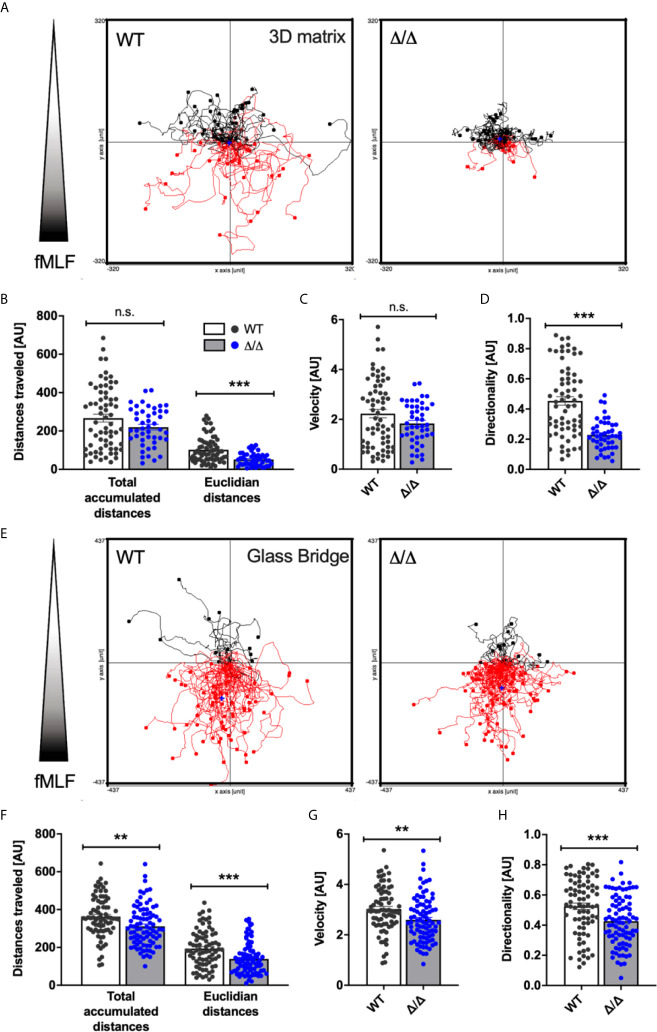
SHIP2 activity is required for chemotactic directionality. Bone marrow-derived wild-type (WT) and Ship2^Δ/Δ^ (Δ/Δ) neutrophils were allowed to chemotax towards 300nM fMLF either embedded in a collagen-matrix in Ibidi chemotaxis μ-slides **(A–D)** or in Dunn chemotaxis chambers **(E–H)**. The orientation of the gradient is indicated to the left of spider plots shown in **(A)** and **(E)** Cell migration was recorded by time-lapse imaging, with pooled tracks of individual neutrophils recorded with cells from three separate preparations were plotted as spider plots **(A, E)** and analyzed **(B–D, F–H)** using the Ibidi Chemotaxis tool plug-in into Image J. Accumulated and Euclidean distances **(B, F)**, Velocity **(C, G)** and Directionality **(D, H)** are plotted. *p* values were determined using the Mann-Whitney test. **p < 0.01; ***p < 0.001; n.s., not significant.

Given the substantial recruitment defect of Ship2^Δ/Δ^ neutrophils *in vivo*, we next examined the involvement of SHIP2 catalytic activity in neutrophil chemotaxis *in vitro*. We allowed wild-type control and Ship2^Δ/Δ^ neutrophils to migrate through a linear concentration gradient of fMLF in a 3D collagen matrix. The tracks of individual neutrophils were plotted in spider plots (**Figure 3A**) and parameters of the tracks, including total accumulated and Euclidian distances travelled, velocity and directionality were calculated. This identified that Ship2^Δ/Δ^ neutrophils were able to migrate in response to the fMLF stimulation. The Euclidian (i.e. the straight line between the start and end point), but not the total distances covered by Ship2^Δ/Δ^ neutrophils were smaller than those of wild-type controls (**Figure 3B**), indicating that directionality, but not the ability to migrate nor the speed of Ship2^Δ/Δ^ neutrophils was reduced (**Figures 3C, D**). We concluded that SHIP2 regulates neutrophil chemotaxis.

Migration in a 3D matrix is integrin-independent, whereas migration on glass is dependent upon integrins (17, 26–28). Since SHIP1 regulates integrin-dependent processes including chemotaxis (13, 15), we also analyzed neutrophil chemotaxis in Dunn chambers (29), where neutrophils migrate in a shallow gradient of chemoattractant on a glass bridge (**Figure 3E**). Again, we observed significant chemotaxis defects as indicated by reduced Euclidian distances covered and reduced directionality by Ship2^Δ/Δ^ neutrophils compared to controls (**Figures 3F, H**). Interestingly, with Dunn chamber chemotaxis the total accumulated distances travelled and the speed of Ship2^Δ/Δ^ neutrophils were also smaller than those of controls (**Figures 3F, G**), suggesting that there may be an additional, integrin-dependent component to the chemotaxis defect conferred by Ship2^Δ/Δ^.

In summary, these experiments highlight that SHIP2 is a regulator of neutrophil chemotaxis.

### SHIP2 Regulates Firm Adhesion Under Conditions of Flow

**Figure 4:**
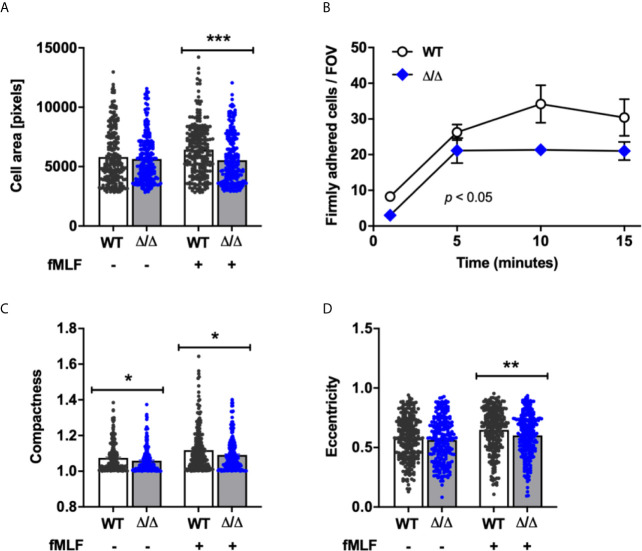
SHIP2 regulates adhesion under flow and chemoattractant induced polarization. Bone marrow derived wild-type (WT) and Ship2^Δ/Δ^ (Δ/Δ) neutrophils were prepared and **(A)** plated onto glass coverslips in the presence of absence of 1 μM fMLF for 5 minutes prior to being fixed. The area of GR1-positive cells obtained from 5 separate neutrophil preparations for a total of 240 cells per condition was measured using CellProfiler software. **(B)** Neutrophils were perfused at constant sheer stress through flow chambers coated with ICAM-1, rmE-selectin and rmCXCL1 as detailed in Materials and Methods. The graph shown combined results obtained from a minimum of three separate experiments. **(C, D)** Neutrophils from five separate neutrophil preparations were plated onto glass coverslips in the presence of absence of 1 μM fMLF for 5 minutes prior to being fixed. Compactness **(C)** and eccentricity **(D)** of GR1-positive cells were analyzed by CellProfiler to determine neutrophil polarization according to compactness (**C**; defined as the mean squared distance of the object’s pixels from the centroid divided by the area, and where a full circle is attributed a value of 1 and larger values are given to irregular shapes), and eccentricity (**D**; the ratio of the distance between the foci of the ellipse and its major axis length, where 0 is a perfect circle, and 1 represents a straight line). *p* values were determined using the Mann-Whitney test **(A, C, D)** or an unpaired, two-tailed t test of the area under the graphs **(B)**. *p < 0.05; **p < 0.01; ***p < 0.001.

To get a better understanding of the extent to which SHIP2 may be required for integrin-dependent neutrophil functions, we next analyzed cell adhesion and spreading. We performed adhesion assays under static conditions, seeding cells onto glass with or without fMLF stimulation, and measured the area of the fixed, adherent neutrophils. While we did not observe any difference in terms of numbers of cells attached under either condition, the mean area occupied by fMLF-stimulated (but not unstimulated) attached Ship2^Δ/Δ^ neutrophils was smaller than that of controls, suggesting a subtle defect in fMLF-induced spreading (**Figure 4A**).

In vivo neutrophils adhere to the vessel wall in the context of blood flow rather than in a static situation. We therefore analyzed neutrophil adhesion of neutrophils to ICAM-1, E-selectin, and CXCL-1 in parallel plate flow chambers. Interestingly and contrasting with the observations in the static adhesion assays, we observed fewer firmly adhering Ship2^Δ/Δ^ compared to wild-type control neutrophils (**Figure 4B**). Together these observations suggest that SHIP2 has a subtle regulatory function in neutrophil adhesion and spreading, which becomes more apparent under conditions of flow.

For neutrophils to migrate directionally in a gradient of chemoattractant, they polarize in response to chemoattractant stimulation. To better understand the reason for the observed directionality defect, we compared the abilities of Ship2^Δ/Δ^ and wild-type control neutrophils to polarize by analyzing two morphological parameters, compactness and eccentricity in response to uniform fMLF stimulation. According to both parameters, stimulated Ship2^Δ/Δ^ neutrophils polarized less efficiently than wild-type controls (**Figures 4D, E**).

### SHIP2 Does Not Regulate ROS Production, Degranulation or Phagocytosis

**Figure 5:**
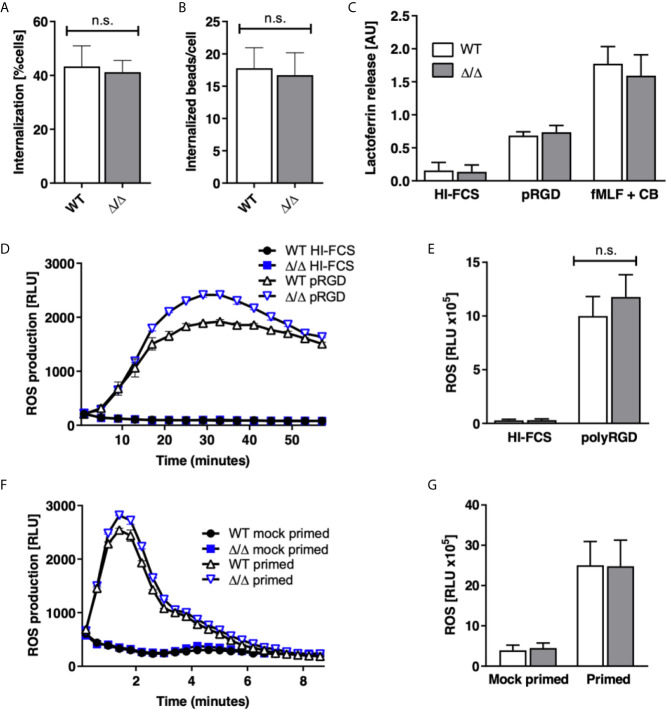
SHIP2 is dispensable for ROS production, degranulation and phagocytosis. Bone marrow derived wild-type (WT) and Ship2^Δ/Δ^ (Δ/Δ) neutrophils were assayed for **(A, B)** phagocytosis of rabbit IgG-opsonized latex beads. Results obtained in 5 separate experiments are presented as bar graphs. **(A)** Percentage of cells that had internalized beads; **(B)** average number of beads internalized per cell. **(C)** Degranulation. Cells were stimulated by being plated onto plastic blocked with heat inactivated (HI) FCS or coated with the pan integrin ligand poly-Arg-Gly-Asp (pRGD), or stimulated with fMLF in the presence of cytochalasin B and lactoferrin release was measured by ELISA. Means ± SEM obtained from 4 separate experiments are integrated in this experiment. **(D–G)** ROS production, with neutrophils stimulated by being plated onto integrin ligands **(D, E)** or with the soluble stimulus fMLF **(F, G)**. **(D, F)** Representative experiments and **(E, G)** accumulated light emission (mean ± SEM) from 4 separate experiments are shown. Pairwise comparison between wild-type and Ship2^Δ/Δ^ neutrophils were not significant under any of the conditions tested. n.s., not significant.

Neutrophils perform a range of effector functions required for killing of pathogens, which include phagocytosis, ROS production and degranulation. We asked whether SHIP2 regulates these functions. Our experiments identified no significant defect in the ability of Ship2^Δ/Δ^ neutrophils to phagocytose IgG-opsonized latex beads in terms of the percentage of cells that internalized beads, nor the number of internalized beads (**Figures 5A, B**).

ROS production and degranulation can be induced by stimulating a number of receptors, an effect that can be useful for establishing whether a regulator acts downstream of a particular receptor. We stimulated neutrophils by plating them onto a synthetic multivalent pan-integrin ligand, polyArg-Gly-Lys, which does not depend on co-stimulation of a second receptor (30), and also with fMLF, but did not detect any significant differences in ROS produced, nor lactoferrin released under any of these conditions (**Figures 5C–G**). Together these results suggest that SHIP2 is not required for the ability of neutrophils to produce ROS or to degranulate in response to stimulation of integrins nor formylated peptide receptors.

### SHIP2^Δ/Δ^ Has No Major Effect on Agonist-Stimulated PKB Phosphorylation or PIP3 Production

**Figure 6:**
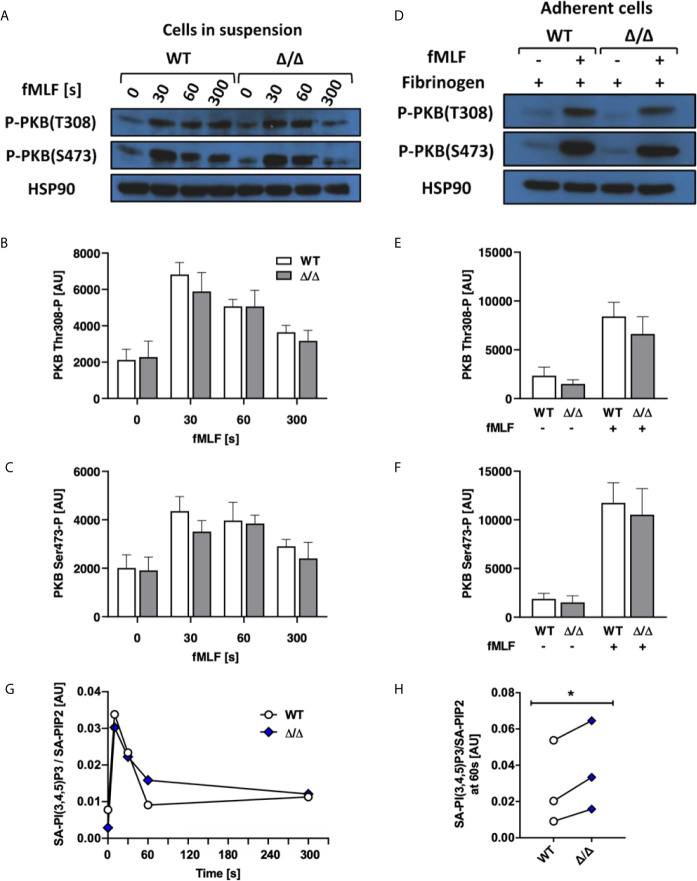
SHIP2^Δ/Δ^ has no major effect on agonist-stimulated PKB phosphorylation or PIP3 production. Bone marrow-derived wild-type (WT) and Ship2^Δ/Δ^ (Δ/Δ) neutrophils were stimulated and **(A–F)** subjected to analysis of PKB phosphorylation. **(A–C)** Cells in suspension were stimulated with 1 μM fMLF at 37°C for the indicated times or **(D–F)** neutrophils were plated onto 150 μg/mL fibrinogen-coated tissue culture plastic in the presence of absence of 1 μM fMLF at 37°C for 19 minutes and processed for Western blotting. Blots were probed for phospho-PKB Thr 308 and Ser 473 with HSP90 as a loading control. Representative blots are shown **(A, D)** and densitometrical analyses combining 5 **(B, C)** or 4 **(E, F)** separate experiments are plotted (mean ± SEM). **(G, H)** Neutrophils were stimulated with 10 μM fMLF for the indicated times at 37°C, and PIP3 generated was analyzed by mass spectrometry. **(G)** A representative experiment, presenting stearoyl/arachidonyl (SA) PIP3 divided by SA-PIP2. **(H)** At the 60 s timepoint, Ship2^Δ/Δ^ neutrophils reproducibly contained subtly increased PIP3. **(G, H)**, Symbols represent individual experiments. Statistical analysis was by 2-way ANOVA with multicomparison post-hoc test **(B, C, E, F)** and a paired t test **(H)**. *p < 0.05.

Stimulated and unstimulated neutrophil lysates from SHIP1-deficient mice were characterized by enhanced PKB Thr 308 and Ser 473 phosphorylation attributed to the increased levels of PI(3,4,5)P3 accumulation (9). To test if this holds true for Ship2^Δ/Δ^ neutrophils, we carried out Western blots to detect PKB phosphorylation as an indirect measurement of PI3K activity, where it is phosphorylated on Thr 308 by PDK1, a direct effector of PI3K and on Ser 473 indirectly *via* mTORC2 (31–33). We performed an fMLF stimulation timecourse, observing no significant differences in PKB phosphorylation of either residue (**Figures 6A–C**). Having determined an integrin-dependent component with functional assays (**Figures 3** and **4**), we also analyzed PKB phosphorylation upon plating neutrophils onto an integrin ligand (fibrinogen) in the presence or absence of co-stimulation with fMLF, but again observed no significant difference between genotypes (**Figures 6D–F**).

Since PKB can associate with PIP3 or PI(3,4)P2 for phosphorylation (34), analyzing its phosphorylation state may not inform on an altered ratio between PIP3 and PI(3,4)P2. For a direct readout, we therefore repeated the stimulation timecourse, and directly quantified PIP3 in fMLF and mock-stimulated neutrophils using mass spectrometry (35). This revealed that PIP3 in fMLF-stimulated Ship2^Δ/Δ^ neutrophils was subtly but significantly increased at one minute after fMLF stimulation compared to wild-type controls (**Figures 6G, H**).

### Ship2^Δ/Δ^ Neutrophils Contain Less PI(3,4)P2 Than Controls

**Figure 7:**
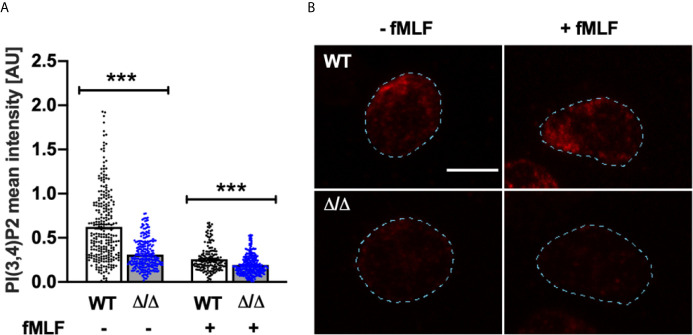
Ship2^Δ/Δ^ neutrophils contain less PI(3,4)P2 than controls. Bone marrow-derived wild-type (WT) and Ship2^Δ/Δ^ (Δ/Δ) neutrophils were plated onto glass coverslips in the presence or absence of 1 μM fMLF for 5 minutes prior to being fixed and subjected to immunostaining with an anti-PI(3,4)P2 antibody. **(A)** PI(3,4)P2 signal intensity was analyzed automatedly using CellProfiler as detailed in Materials and Methods. The graph presented combines cells from 3 separate experiments for a minimum of 176 cells per condition and data were analyzed with the Mann-Whitney test. ***p <0.001. **(B)** Representative examples of PI(3,4)P2-stained neutrophils for each condition. For ease of viewing, the outline of the cells shown here was traced using FiJi (broken lines). Scale bar, 5 μm.

The functional differences we observed between Ship2^Δ/Δ^ and wild-type control neutrophils could be due to the minor change in global PI(3,4,5)P3 that we had observed (**Figure 6H**). Alternatively, it could be due to changes in cellular PI(3,4)P2, a second messenger in its own right. We performed mass spectrometry to measure this minor phosphoinositide species (23), but unfortunately this approach was not sufficiently sensitive to detect changes in PI(3,4)P2 in response to fMLF stimulation even with control mouse neutrophils (data not shown). We therefore resorted to immunofluorescence, making use of a PI(3,4)P2 antibody to analyze this phosphoinositide in adherent neutrophils. We noticed that PI(3,4)P2 predominantly resided on neutrophil endomembranes, consistent with its function in endocytic processes (36–38). Interestingly, when analyzing fluorescence intensity of control and Ship2^Δ/Δ^ neutrophils that had or had not been stimulated with fMLF while being allowed to adhere to glass coverslips, we observed significantly less PI(3,4)P2 signal in Ship2^Δ/Δ^ than in wild-type neutrophils under both of these conditions (**Figures 7A, B**). Hence loss of SHIP2 catalytic activity caused reduced cellular PI(3,4)P2 of Ship2^Δ/Δ^ neutrophils at least in this context which analyzed adhesion coupled with fMLF stimulation.

## Discussion

This study characterized the function of SHIP2 in the neutrophil, analyzing neutrophils isolated, and bone marrow chimeras generated from a mouse carrying a homozygous Ship2^Δ/Δ^ that contained a small deletion in the catalytic domain, which rendered SHIP2 catalytically dead. Unlike with Ship2-deficiency, analysis of Ship2^Δ/Δ^ neutrophils allowed us to identify neutrophil functions that were dependent on SHIP2 catalytic activity without being confounded by potential scaffold effects, although the major phenotypes of Ship2^-/-^ and Ship2^Δ/Δ^ mice were very similar (16, 25). It is possible that compensatory events reduced the severity of the phenotype we observed, and that use of an inducible, rather than a germ-line Cre to generate Ship2^Δ/Δ^ mice might have resulted in a more severe phenotype.

Despite these considerations, we identified a clear-cut role for this 5-phosphatase in regulating neutrophil directionality during chemotaxis *in vitro* (**Figure 3**) together with a substantial defect in neutrophil recruitment to sites of sterile inflammation *in vivo* (**Figure 2**). We further observed a defect in firm adhesion under flow with neutrophils that were simultaneously stimulated with immobilized integrin ligand, chemokine and selectin and subtle defects in neutrophil polarization and spreading in response to uniform chemoattractant stimulation (**Figure 4**). In contrast, no significant defects were observed with any other neutrophil functions tested (phagocytosis, degranulation, ROS production; **Figure 5**). Mechanistically, we conclude that the phenotype of Ship2^Δ/Δ^ neutrophils is largely due to reduced cellular PI(3,4)P2 (**Figure 7**) rather than globally increased PIP3.

A large body of work has implicated PI3Kγ and δ isoforms in the regulation of neutrophil chemotaxis/chemokinesis *in vitro* and recruitment to inflamed sites *in vivo*, with some later studies suggesting a context-dependent function (4, 5, 39–45). While the 3-phosphatase PTEN appears to have a rather subtle regulatory function in neutrophil chemotaxis (10, 15, 46), neutrophils deficient in the 5-phosphatase SHIP1 are characterized by excessive adhesion and spreading, defects in polarization (when adherent) and chemotaxis *in vitro* as well as hyperactivity in ROS production induced by integrin ligation (13, 15). Our finding of impaired chemotactic directionality caused by loss of SHIP2 catalytic activity complements this and suggests important non-redundant regulatory functions of the two 5-phosphatases in neutrophil chemotaxis and recruitment.

Contrasting with SHIP1-deficient neutrophils, which were characterized by substantially increased PIP3 production (15), there was only a very subtle increase in PIP3 with Ship2^Δ/Δ^ neutrophils that had been stimulated for 60s with fMLF (**Figure 6**). Rather we observed substantially reduced intracellular PI(3,4)P2 in cells that were allowed to adhere to glass in the presence and absence of fMLF (**Figure 7**). PI(3,4)P2 at endomembranes has been attributed to Class II PI3K 2a-dependent phosphorylation of PI4P in the context of clathrin-dependent endocytosis (36), but also to 5-dephosphorylation of PIP3 in clathrin-independent endocytic processes, where SHIP2 has been implicated (37, 38, 47). Overall our data suggests distinct functions of SHIP1 and SHIP2 in the neutrophil which together control neutrophil chemotaxis and recruitment. Given that variation in housing conditions and microbiota regulate neutrophil production and functions (48) and that discrepancies in experimental conditions can differentially modulate neutrophil activation status, it is possible that a side-by-side comparison of both lines may have unearthed additional features which were missed here.

Still, altogether our work suggests that both SHIP family 5-phosphatases are important regulators of neutrophil functions. Hence SHIP2 specifically regulates chemotactic directionality and neutrophil recruitment to sites of inflammation, while SHIP1 is a regulator of adhesion-dependent neutrophil functions. As our understanding of the wider family of 5-phosphatases grows (49), we will continue to learn how this group of enzymes regulates different facets of neutrophil biology.

## Data Availability Statement

The original contributions presented in the study are included in the article/**Supplementary Material**. Further inquiries can be directed to the corresponding author.

## Ethics Statement

The animal study was reviewed and approved by University of Edinburgh Animals Welfare and Ethical Review Body.

## Author Contributions

MM performed *in vitro* experiments, data analysis, and wrote the paper. BM and UK performed *in vivo* experiments and data analysis. MV helped with image acquisition and analysis. KA performed mass spec and data analysis. SS provided Ship2^Δ/Δ^ mice. AA provided resources and supervision. SV conceived the study, performed *in vitro* experiments, data analysis, supervision and wrote the paper. All authors edited and concur with the paper. All authors contributed to the article and approved the submitted version.

## Funding

MM was funded by a BBSRC iCASE studentship with GSK (BB/R505651/1). BM and SV were funded by the MRC (MR/M023060/1) and UK holds a Versus Arthritis PhD scholarship (21577).

## Conflict of Interest

AA is employed by GlaxoSmithKline.

The remaining authors declare that the research was conducted in the absence of any commercial or financial relationships that could be construed as a potential conflict of interest.
