# Supplementary material for: The 5-Phosphatase SHIP2 Promotes Neutrophil Chemotaxis and Recruitment
Source: Front Immunol. 2021 Apr 19;12:671756. doi: 10.3389/fimmu.2021.671756 (PMC8089392; doi:10.3389/fimmu.2021.671756)
Supplement: Supplementary file 1 [file Image_1.pdf]

Supplemental Material

Michael et al, The 5-phosphatase SHIP2 promotes neutrophil chemotaxis and recruitment

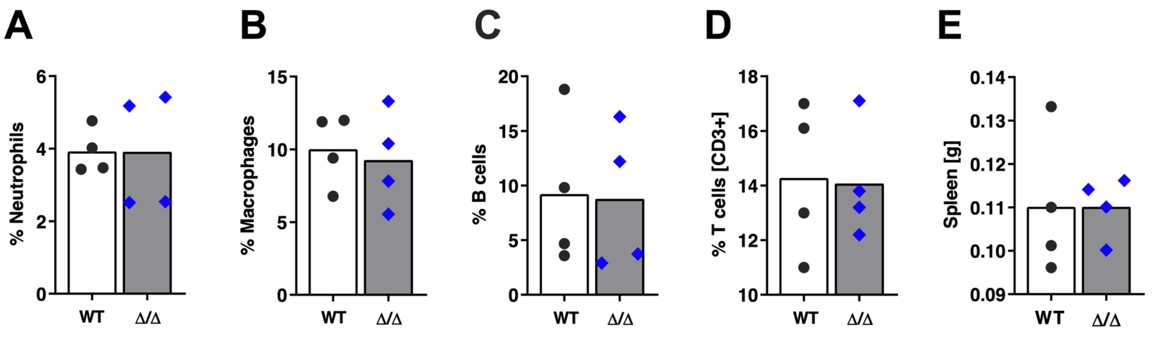

Figure S1. Lungs of Ship2$^{\Delta/\Delta}$ mice are not infiltrated by leukocytes. Unchallenged 7-9 month-old wild-type (WT) and Ship2$^{\Delta/\Delta}$ (Δ/Δ) mice were sacrificed. Lungs were perfused through the right ventricle prior to being dissected and spleens were dissected. Single cell lung digests were gated for CD45-positive singlets; percentages of CD45-positive immune cells are plotted; neutrophils (LY6G-high; A), monocytes/macrophages (F4/80 and CD64-positive; B), B cells (B220-positive; C) and total T-cells (CD3 positive; D). (E) Weight of spleens. Each symbol represents one mouse and the bars show the mean. *p* values were calculated using unpaired two-tailed *t*-tests.
